# In dogs diagnosed with osteoarthritis, how safe and effective is long-term treatment with bedinvetmab in providing analgesia?

**DOI:** 10.18849/ve.v8i1.598

**Published:** 2023-02-01

**Authors:** Katrin Kronenberger+

**Keywords:** CANINE, OSTEOARTHRITIS, BEDINVETMAB, MONOCLONAL ANTIBODY, CANINE NERVE GROWTH FACTOR, ANALGESIA, CHRONIC OSTEOARTHRITIS ASSOCIATED PAIN, SAFETY, EFFECTIVENESS

## Abstract

There is an erratum to this paper published in Veterinary Evidence Vol 8, Issue 3 (2023): https://doi.org/10.18849/ve.v8i3.668

**PICO question:**

In dogs diagnosed with osteoarthritis (OA), how safe is treatment with bedinvetmab, when compared to a placebo and how effective in long-term reduction of the severity of the clinical signs associated with OA-related pain?

**Clinical bottom line:**

**Category of research:**

Treatment.

**Number and type of study designs reviewed:**

Two papers were critically reviewed. One was a randomised, blinded, multi-arm laboratory safety study; the other a multi-center field trial consisting of a block-randomised, double blind, placebo-controlled phase, followed by a non-comparative, open-label case series study.

**Strength of evidence:**

Weak.

**Outcomes reported:**

One study rated the reduction of the severity of clinical signs associated with OA pain using owner Canine Brief Pain Inventory (CBPI) ratings and non-validated veterinary clinical assessments (VCAs). Safety was addressed by evaluating adverse health effects (AHEs), concentration of antidrug antibodies (ADAs), and clinical pathology. Significant improvements in pain scores and VCAs were reported in this treatment study. The second safety-only study used clinical observation to evaluate AHEs. Both studies reported AHEs.

**Conclusion:**

The quality of the published evidence available to answer the PICO question ‘In dogs diagnosed with osteoarthritis, how safe and effective is long-term treatment with bedinvetmab in providing analgesia’ is weak due to design limitations of the two studies so far published. The decision to use bedinvetmab remains dependent on the judgement and experience of the clinician.

## How to apply this evidence in practice

The application of evidence into practice should take into account multiple factors, not limited to: individual clinical expertise, patient’s circumstances and owners’ values, country, location or clinic where you work, the individual case in front of you, the availability of therapies and resources.

Knowledge Summaries are a resource to help reinforce or inform decision making. They do not override the responsibility or judgement of the practitioner to do what is best for the animal in their care.

## Clinical scenario

A 12-year-old Laika-cross has been diagnosed with canine osteoarthritis (OA), affecting his coxofemoral and stifle joints.  Meloxicam helped manage the dog’s pain for over 12 months successfully. However, the owner is concerned about the risk of adverse effects and asks for alternative treatment options not involving non-steroidal anti-inflammatory drugs (NSAIDs). You are considering bedinvetmab (Librela, Zoetis), which has only recently become available. You want an understanding of efficacy, effectiveness, and safety evidence before deciding whether this is an alternative treatment option to suggest to the owner.

## The evidence

The Krautmann et al. (2021) pre-clinical laboratory study addressed the safety effects of bedinvetmab in healthy, mature laboratory beagles. The authors neither describe the process of random assignment to the three study groups, nor further assignment to the smaller subgroups within these separate studies. However, the authors make a general statement at the beginning of the methods section that good laboratory practice guidelines were followed. In this study, dogs were stratified by sex. The small cell sizes (n = 8) within each study condition raise concerns of overall potential sample bias or cell-specific sample bias. Sample size of licensing studies is covered in internationally harmonised recommendations which try to balance risks of small sample size leading to potential bias with minimising the use of experimental animals (3Rs). Whilst safety studies are designed as far as possible to be applicable across the whole population, the genetic diversity, intercurrent disease and other drugs patients may be receiving cannot be covered. For these reasons, field studies are conducted and ongoing adverse event reporting instituted to identify subpopulations that may be at risk.

The Corral et al. (2021) ‘change from baseline’ study assessed both efficacy and safety of bedinvetmab in client-owned dogs with OA. Pretreatment Canine Brief Inventory (CBPI) pain scores were used as baseline comparison and included in the efficacy analysis of bedinvetmab. Standardisation instructions on using the CBPI explicitly indicate that the first CBPI score should be discarded due to potential regression to the mean (RTM) (Brown et al., 2008). It is unclear whether Corral et al. (2021) followed the guidance to use the second pain score as baseline, which could result in treatment and control groups being significantly different. Interpretation of this difference was confounded by 7 days of bedinvetmab treatment preceding this second score. This multi-site, multi-national trial spanned several countries with different numbers of sites in each country and different numbers of dogs at each site. The CBPI was not translated into the target languages, potentially impacting its reliability / validity. Loss to follow-up affected placebo and treatment groups differentially, which may increase the risk of bias. 22/146 (15.1%) of the dogs randomised to the placebo group, and 9/141 (6.4%) of the dogs randomised to the bedinvetmab group were excluded from the efficacy analysis. Loss to follow-up and blinding was incomplete.

The quality of the published evidence available to answer the PICO question is weak due to design limitations of the two studies so far published. Further studies are required to better understand long-term efficacy and short-term safety across the patient population.

## Summary of the evidence

**Corral et al. (2021):** 

Population:	Client–owned dogs of any breed, sex, and body weight. Eligibility criteria for inclusion: Dogs aged ≥ 12 months at enrollment. Osteoarthritis (OA) in at least one joint of the pelvic or thoracic limbs (confirmed by orthopaedic examination and supported by radiographic evidence). Canine Brief Pain Inventory (CBPI): dogs with an initial pain severity score (PSS ≥ 2) and a pain interference score (PIS ≥ 2) (owner-assessed) were confirmed eligible. Orthopaedic examination: dogs with at least one of three components of the veterinary clinical assessment (VCA) assessed as ‘moderately affected’ on day 0 (1. Lameness / weight-bearing; 2. pain on palpation / manipulation of joint(s); 3. general musculoskeletal condition), were confirmed eligible. No other uncontrolled concurrent disease or concomitant treatment, and satisfactory blood and urine clinicopathological results. Criteria for exclusion: Enrollment in a clinical trial of any type ≤ 30 days prior to day 0. Previous treatment with an anti-nerve growth factor monoclonal antibody (anti-NGF mAb). Pregnancy, lactating or intended use as a breeding animal. Anticipated surgical intervention, enrollment in physical therapy or in a weight loss program < 8 weeks before day 0. Lameness known to be related to neoplasia, primary neurologic or immunologic disorder, infection, recent joint trauma, or non-healed fracture. History of intervertebral disc disease or evidence of injury resulting in neurologic deficits. Administration of any of the prohibited medications.
Sample size:	287 dogs. Initial 3 month randomised, controlled, blinded comparative phase: Client-owned dogs diagnosed with osteoarthritis (n = 287) enrolled from 26 veterinary practices across Europe (Hungary [n = 8], Ireland [n = 6], Germany [n = 3], Portugal [n = 9]). n = 22 dogs were removed requiring rescue treatment before day 84: n = 19 in the placebo group and n = 3 in the treatment group. 6 month single-armed, open-label continuation phase: A non-randomised subset of bedinvetmab-treated dogs with a ‘positive response’ to treatment (n = 89) enrolled from 14 veterinary practices across Europe (Portugal [n = 5], Hungary [n = 4] and Ireland [n = 5]). n = 11 dogs were removed before study completion; n = 10 developed unrelated medical conditions and one case required rescue treatment. Reported numbers of dogs in both phases of the study do not seem to match reported number of dogs at start and after rescue removal. n = 23 dogs (8.01%) were removed from the study, mainly due to worsening of clinical signs of OA. Sample size estimates (≥ 120 dogs / group) were derived from power calculations based on variance and effect sizes from unpublished data. Aim was to achieve at least 80% power at a significance level of ɑ = 0.05 (two-sided). CBPI pain scores PSS and PIS were analysed using a generalised linear mixed model for repeated measures. Pre-treatment scores (baseline) were used as covariates; treatment and interaction between treatment and day of study were used as fixed effects in the model. Random effects included site, block within site, interaction between site, treatment and day of study, and error. Primary outcome response variables are binary (treatment success yes / no) and were analysed using a generalised linear mixed model with binomial distribution and logit link with level of significance set at ɑ = 0.05 (two-sided). Fixed effect was treatment and random effects site, block within site, and interaction between site and treatment. Back-transformed least square mean (LSM) proportion, 95% confidence interval (CI) and standard error (SE) were used to report the estimated proportion of dogs having achieved treatment success.
Intervention details:	Intervention Bedinvetmab (Librela, Zoetis) dosage between 0.5–1.0 mg/kg was administered subcutaneously (SC) monthly for a maximum of 9 months. Control 0.9% saline was administered SC at a dose volume equivalent to bedinvetmab administered monthly for 3 months. Initial 3 month randomised, controlled, blinded, comparative phase: Blocked randomisation: Two eligible dogs per block were assigned at random to the placebo (n = 146) or the bedinvetmab group (n = 141) ensuring an allocation ratio of 1:1 at each test site in order of entry, based on a randomisation protocol developed by a statistician. A dispenser used an electronic data capture system to randomise the dogs. Blinding: Owners and all study personnel were blinded to group assignment, apart from the treatment dispenser at each test site, who was responsible for preparation and administration of study treatment. Pretreatment data were collected at enrollment and used as baseline for analysis. First treatment administration on day 0. Dogs were examined and samples collected for haematology, serum chemistry and urinalysis during seven visits on days 0, 7, 14, 28, 42, 56 and 84. Dosing occurred on days 0, 28 and 56. 6 month single–armed, open–label, uncontrolled continuation phase: Bedinvetmab–treated dogs who had ‘responded positively’ to treatment (n = 89) enrolled; 78 (n = 78) completed seven additional monthly visits over 6 months. During each visit: Owners completed the CBPI. A veterinarian performed a physical examination and completed a VCA. Blood samples were collected (haematological variables, serum chemistry, bedinvetmab, total NGF serum concentration and anti-drug antibodies (ADAs) analysed. Every 3 months urine was collected for urinalysis and evaluation of protein creatinine ratio. Dogs could be withdrawn at any time by owner or veterinarian. After exiting the study, dogs could resume conventional OA treatment. In case of worsening clinical signs of OA or perceived lack of efficacy (LOE), a rescue treatment i.e., a prohibited treatment, was considered and the dog defined as ‘not having improved’.
Study design:	A randomised, double-blind, placebo-controlled, parallel-arm, multi-site experimental field study, followed by a 6 month single-arm, open-label case series study.
Outcome studied:	Primary efficacy outcome measure (subjective) CBPI-based treatment success was defined as a reduction of ≥ 1 in the pain severity score (PSS) and of ≥ 2 in the pain interference score (PIS) on day 28 compared with owner-assessed pretreatment (baseline) PSS and PIS scores. Secondary efficacy outcome measures (subjective) CBPI treatment success for all other assessed time points. Owner-assessed PSS and PIS scores (CBPI) for all other time points. Owner-assessed overall impression of quality of life (QoL). Percentage of dogs having a ‘very good’ or ‘excellent’ life at each time point. Assessment of the overall improvement of the VCA across the three components compared to baseline a) improved in at least one component and the others not worse and b) improved scores in at least two components and the other score worse or unchanged. A dog was defined as ‘not having improved’ if neither a or b could be applied, or if a dog had been withdrawn because of perceived LOE or had received rescue treatment. Safety outcome measures (objective) All animals enrolled in both phases were included in the safety data analysis. Phase 1: placebo group n = 146 and bedinvetmab group n = 141; Phase 2: n = 89 bedinvetmab-treated dogs. Frequencies of dogs with at least one adverse health event (AHE) were summarised by clinical sign and clustered in organ classes. Clinical haematology and urinalysis: reference ranges were compared to baseline and between treatment groups. Immunogenicity data (development of ADAs) were evaluated by integrating the ADA data with bedinvetmab and total NGF concentrations.
Main findings:	Efficacy of bedinvetmab A significantly greater proportion of dogs in the bedinvetmab group 58/133* (43.5%) achieved CBPI-based treatment success versus placebo group 22/137* (16.9%) on day 28 (P = 0.0017). The difference between the groups is statistically significant (the null hypothesis that treatment with bedinvetmab is no different to treatment with placebo can be rejected). The clinical impact of the treatment success is difficult to assess in the absence of appropriate size estimates and confidence intervals. The difference on day 28 of the pain severity score (PSS) least squares mean is approximately 0.9 (on the 10 point scale) and of the pain interference score (PIS) approximately 1.2 (on the 10 point scale). Criteria for success for an individual compared to their baseline data was set at a reduction in PSS score of ≥ 1 and PIS of ≥ 2. Maximum bedinvetmab treatment effect was observed on day 42: 70/134* (52.6%) of bedinvetmab treated dogs versus 29/140* (21.1%) of dogs in placebo group (P = 0.0001). Mean PSS and PIS scores (CBPI) on all other days were significantly different between bedinvetmab and placebo groups (p ≥ 0026). The percentage of dogs that demonstrated improvement in the CBPI overall impression of QoL was higher in the bedinvetmab group than in placebo group at every visit during the comparative phase (no data provided). VCA improvement versus baseline was significantly different in bedinvetmab versus placebo group (data not shown; p <01). Overall improvement based on VCA was significantly different in the bedinvetmab group (69.2–91.4%) versus the placebo group (≤ 9%) (p ≤ 0.0002). Safety of bedinvetmab Most frequently reported AHE was joint pain and lameness (23/146 [15.8%] dogs in the placebo group and 5/141 [3.5%] dogs in the bedinvetmab group). Serious AHEs: two dogs died during comparative phase; four dogs died during continuation phase. Deaths were considered unrelated to treatment. n = 25/146 (17.1%) dogs in the placebo and n = 11/141 (7.8%) dogs in the bedinvetmab group received anti-inflammatory and antirheumatic concomitant medication, which was well tolerated and not associated with AHEs in the bedinvetmab group. Dogs in the bedinvetmab group showed a decrease in haemoglobin (Hb) and packed-cell volume haematocrit (PCV) levels and an increase in aspartate aminotransferase and blood urea nitrogen concentrations compared to baseline and reference ranges and compared to dogs in the placebo group. A mild transient reaction at injection site was observed in one dog and was resolved within 6 and 7 days. A total of four dogs developed treatment-emergent ADAs during study (two transient and two persistent). 2/138 (1.4%) dogs developed bedinvetmab-associated immunogenicity; clinical manifestation was reduced efficacy of treatment (CBPI treatment success was not achieved at most time points). *These values were generated by the Knowledge Summary author and based on the back transformed mean proportions reported.
Limitations:	It is unclear whether Corral et al. (2021) followed the CBPI guidance to use the second pain score as baseline. If the second score was not used, this could result in treatment and control groups being significantly different. CBPI administration violations may have resulted in initial differences between treatment and control groups. First baseline comparison was significantly different. This produces a high level of uncertainty when associating the reported changes of pain scores in bedinvetmab treated dogs with the clinical meaningful reduction of pain. Removing randomised dogs’ outcome data from the efficacy analysis may inflate the estimated treatment effect. However, the potential effect of removing these data on the final analysis would not have changed the overall outcome. Randomised dogs’ outcome data were removed from the efficacy analysis but were included in safety analysis, which followed the study protocol but could lead to biased results of unknown direction. Statistical management of missing data was not discussed. No information on how the study was presented to owners prior to enrollment. No information on whether there was bias about which owners received study information. CBPI was not translated into target languages, which may impact its reliability and validity. VCA was not validated, and data was not presented. A decrease in Hb and PCV after administration of bedinvetmab was not discussed, and data not presented despite the potential for unexpected haematologic effects associated with mAb therapies. The lack of inclusion of a placebo control group in the 6 month continuation phase affects internal and external validity of results (acknowledged by the authors), however, long-term treatment with a placebo of dogs with OA would have been unethical. Enrolling a non-randomised subgroup of dogs based on outcome variables obtained in phase 1 of the trial may lead to biased results. No information was provided on how the dispenser’s activity was isolated from veterinary staff or owners, making it difficult to make an assessment of adequate blinding. However, the authors make a general statement in the methods section that good clinical practice guidelines were followed. It is not clear whether randomisation was centralised across the different clinics to minimise selection bias and ensure balance of treatment group factors at baseline. Different sections of the paper provide differing numbers of dogs for in-comparison groups. This presents challenges in ascertaining the accuracy of whether cited p-values are derived from the correct number of subjects or if errors are present. A treatment success (< 25%) in the placebo group based on CBPI owner assessment was not discussed.

**Krautmann et al. (2021):** 

Population:	Criteria for inclusion Clinically healthy, purpose-bred, mature laboratory Beagle dogs; age: 10–12 months old; bodyweight: 5.1–12.7 kg; sexually intact; previously immunised against standard canine pathogens. Criteria for exclusion No details provided.
Sample size:	n = 96 dogs.
Intervention details:	Overall intervention Bedinvetmab (Librela, Zoetis) 15 mg/mL or 30 mg/mL (refrigerated, 2–8 °C), administered subcutaneously (SC) at marked locations on the lateral neck. Control 0.9% sterile saline solution for injection (Hospira, Inc.) administered SC at marked locations on the lateral neck and at volume equivalent to the 10 mg/kg dose volume. Three sub-studies Study 1 (dose-dependent pre-clinical safety evaluation) Maximum intended label dose of bedinvetmab 1 mg/kg. 3 mg/kg and 10 mg/kg overdoses for evaluation of therapeutic and super-saturating overdoses. Bedinvetmab at 10mg/kg is 10x the recommended treatment dose which is twice as much as recommended (EMEA VICH Topic GL43, 2008). Study 2 (evaluation of T-lymphocyte-dependent immune response of bedinvetmab to keyhole limpet hemocyanin (KLH)) KLH: an unadjuvanted subunit, good manufacturing practices (GMP) grade KLH (Stellar Biotechnologies) endotoxin-free (0 EU/mg of protein), formulated in 10 mM phosphate–buffered saline at potencies of 0.1 or 1 mg/mL/dose, and administered intramuscular (IM) into the hindquarter within 24 hours of formulation. KLH was used to examine cellular and humoral immune function by measuring T-cell dependant antibody titres (TDAR test) following treatment with bedinvetmab or placebo. Study 3 (evaluation of adverse effects of concurrent administration of bedinvetmab and NSAID carprofen) 4 mg/kg carprofen (Rimadyl, Pfizer) administered SC daily for 14 days. For each separate study 32 dogs were randomly selected and assigned to pens during the acclimation period (prior to study day 0) and to one of four treatments (n = 8; four males, four females per treatment) in pen order. Randomisation to pens, treatments, and procedures were generated using SAS release 9.4 (SAS Institute). Personnel conducting subjective treatment-phase observations were blinded to group assignments. General, bone / joint, and clinical pathology, and toxicokinetics / ADA data were summarised by respective experts unblinded to other study findings. Acclimation period approximately 1 month prior to dosing. At conclusion dogs were either released back to the stock colony (study 2) or humanely euthanised (studies 1 and 3) using intravenous (IV) sodium pentobarbital (Socumb, Henry Schein) prior to necropsy. Study 1 n=32 split into four treatment groups of n = 8 (four male / four female). Three bedinvetmab dose groups (1 mg/kg, 3 mg/kg, 10 mg/kg) and one saline group. Treatment was administered SC every 28 days for seven doses (6 months duration). Necropsy on days 182/183. Four dogs were ‘deemed unsuitable’ (no further explanation was provided) during the acclimation period and were replaced with dogs of the same sex. Bodyweights by treatment group and sex [Least square mean (LSM), confidence interval (CI) 90%. Day 1 pretreatment baseline. Study 2 n = 32 split into four treatment groups of n = 8 (four male / four female). Two paired groups were allocated to receive 0.1 mg or 1 mg KLH intramuscular (IM); within each pair, one received bedinvetmab 1 mg/kg and the other received saline SC on day 0. During the acclimation period two dogs (one male and one female) were removed due to thin body condition score and requiring dietary changes. They were replaced with two dogs of the same sex. Elisa data was analysed using the average of duplicate background-corrected density data with < 25% coefficient of variation to detect a difference in mean log KLH titres of two or four with 80% power at ɑ = 1. Least square mean (LSM) ± standard error (SE) anti-KLH antibody titres after immunisation at two KLH antigen doses in adult dogs treated with bedinvetmab. Study 3 n = 32 split into four treatment groups of n = 8 (four male / four female). Two groups received saline SC and two groups received bedinvetmab (1 mg/kg SC) on day 0, followed by saline or carprofen (4.4 mg/kg SC) daily for 14 days, followed by necropsy. n = 32 dogs survived until scheduled necropsy. For each study All treatments were administered once on the study days indicated. Dogs in studies one and three had no significant radiographic evidence of pre-existing joint disease.
Study design:	Randomised (stratified by sex), blinded, multi-arm, parallel-group safety study.
Outcome studied:	Study 1 Primary outcome (objective): Pharmacokinetic profile of bedinvetmab: Mean bedinvetmab serum concentrations (μg/mL) after doses one and six, at three dose levels (1 mg/kg, 3 mg/kg, or 10 mg/kg SC); n = 8 per dose group). Study 2 Primary safety endpoint (objective): Effect of bedinvetmab on T-lymphocyte-dependent immune function was evaluated by measuring anti-KLH immunoglobulin (IgG)antibody titers using Elisa (T-cell antibody response). Study 3 Primary safety endpoint (objective): Adverse effects in joints of the appendicular skeleton or organs after 2 weeks of concurrent administration of bedinvetmab and carprofen. Pretreatment radiographs. Objective safety endpoints (studies 1 and 3) Serial clinical observations (injection site, neurological and musculoskeletal examinations). Clinical pathology: sampling included capillary beds, peripheral nerves, immune and joint tissues of the appendicular skeleton and tissues where bedinvetmab: NGF or anti–drug antibody (ADA) complexes might initiate local inflammatory responses. Terminal pathology: absolute / relative organ weights. Radiography of joints.
Main findings:	Study 1 Bedinvetmab administered SC monthly was well tolerated in healthy laboratory dogs at all dose levels. Study 2 The antibody response to KLH was unaffected by exposure to bedinvetmab. Bedinvetmab concentrations and toxicokinetics indicated that clearing or neutralising ADAs were not induced. One dog was euthanised approximately 4 weeks after study completion (7 weeks post-treatment) due to vomiting, diarrhoea, lethargy, anorexia, and fever. Based on histopathology and clinical evaluation this appeared to be unrelated to treatment manipulation. Study 3 Daily co-administration for 14 days of bedinvetmab and carprofen was well tolerated: Gross and microscopic pathology findings in all tissues were incidental and unrelated to bedinvetmab or carprofen. All joints, ligaments, menisci, bone, and marrow were unremarkable. Studies 1 & 3 In selected dogs, local reactions at the injection site (inflammation, heat, swelling) were observed ‘sporadically’ (no semi-quantitative or quantitative evaluation was provided). Statistically significant changes compared to controls were reported for selected parameters in blood and urine samples at some time points and categorised by sex (collected after animals were fasted overnight), but no treatment-related effects were identified in clinical pathology parameters. No meaningful treatment-related changes were identified between pretreatment and pre-terminal survey radiographs of major joints. No ADAs were detected.
Limitations:	Enrolment of healthy, mature dogs at a single site cannot evaluate potential risks across all subpopulations of patients that may receive bedinvetmab. Allocation concealment is not described leaving the possibility of selection bias. However, the authors make a general statement at the beginning of the methods section that good laboratory practice guidelines were followed. Results reporting in some areas would benefit from clarification which would have aided assessment of the data. Intermittent mild inflammation (swelling, heat, or redness) observed at the injection site was not quantified. Multiple concurrent hypothesis testing / pairwise comparison is not detailed. Authors are all employees of the manufacturer of the drug, which is declared in the conflict of interest statement. Personnel conducting subjective treatment-phase observations were blinded to group assignment, however general bone / joint, and clinical pathology, and toxicokinetics / ADA data were summarised by respective experts unblinded to other study findings, potentially introducing bias. Limited information on statistical analysis. Although a 10x dose potentially increases subject risk, the lack of adverse events at this level of overdose supports the safety claims of the study. Study 3 lacks internal (duration and number of dogs involved) and external validity (the dogs used in this study were healthy and young). Whilst concurrent treatment increases difficulty identifying safety concerns related to the test drug i.e. did the test drug affect the side effect rate of the non-steroidal drug or vice versa; it was a stated aim of Study 3. The effect of excluding and replacing dogs from the study on results was not discussed. It is unclear who administered the treatment and whether they were blinded. The significance level set at 10% together with small cell sizes increase the risk of incorrect rejection of the null hypothesis. However, given the small cell size, this significance level is set to avoid missing a true treatment effect i.e. more sensitive but less specific to treatment effect.

## Appraisal, application and reflection

All authors in the Krautmann et al. (2021) study and the majority of authors in the Corral et al. (2021) study are employees of the manufacturer of the drug, which is acknowledged in the author list and declared in the conflict of interest statement. Bedinvetmab (Librela, Zoetis) is a canine immunoglobulin G2 (IgG2) monoclonal antibody (mAb) intended for the alleviation of osteoarthritis-related pain. Its mode of action differs from cyclooxygenase inhibiting NSAIDs, that block the production of prostaglandins. Bedinvetmab modulates nociceptive and neuropathic pathways by targeting and binding to canine nerve growth factor (NGF), blocking NGF / tropomyosin receptor kinase A receptor (TrkA) signaling (Enomoto et al., 2019). Species-specific anti-NGF mAb treatments may have fewer adverse health events than NSAIDs due to their high binding specificity and, unlike opiates, may not block protective nociceptive sensation (Enomoto et al., 2019).

The Corral et al. (2021) multi-centre field trial examined the efficacy and safety of 9 monthly subcutaneous injections (SC) of bedinvetmab (0.5–1.0 mg/kg Librela, Zoetis) in client-owned dogs diagnosed with osteoarthritis (OA). The initial 3 month study was randomised, blinded, and had a placebo control group. The process of randomisation across multiple sites in different countries with different numbers of subjects is unclear. The authors do not describe individual site blinding methodology and do not discuss statistical management of missing data, particularly methods for handling the implementation of rescue medication (Donders et al., 2006). Furthermore, incomplete follow–up minimised data interpretability.

The 6 month, open-label, observational case series study following the 3 month randomised controlled trial (Corral et al., 2021) observed efficacy and safety outcome measures without a control group. The study cited ethical concerns with a 6 month placebo comparison group in a clinical sample. A case series study without control can provide suggestive information about treatment and safety outcomes (i.e., potential serious adverse events), but there is an inherent potential for various biases and confounds. The lack of blinding of outcome assessors was an additional issue with this follow-on. No information was provided about demographics, age or weight, or severity of condition for the dogs selected for follow-on (described as ‘responded positively’ previously to treatment with bedinvetmab). Corral et al. (2021) neither discuss the implications of selecting the second group on this basis, nor define what ‘responding positively’ means.

The study’s primary efficacy outcome measure was owner-assessed OA-related change of pain scores using the Canine Brief Pain Inventory (CBPI) (Corral et al., 2021). Standardisation of the CBPI reports robust statistical power and reliability for  quantifying  owner perception of pain severity and impact of chronic OA-associated pain on dogs’ quality of life (QoL) Brown et al., 2007; 2008; and 2013). Corral et al. (2021) did not adhere to scale administrative guidelines (Brown et al., 2008) by collecting data at enrolment and using these as the baseline for comparison with scores obtained at later time points, despite standardisation guidance to avoid using scores collected on the first appointment given concerns about regression (Brown et al., 2008; and Friedman et al., 2015). A measurable subject variable, like pain, varies over time. Pain scores that are 'extreme' by chance tend to 'regress towards the mean' when repeated. The natural variation in repeated in-subject measurements may erroneously infer clinically meaningful change after intervention and affect clinical decisions, while regression to the mean (RTM) confounded the change from baseline (Barnett et al., 2005). Beginning baseline data collection after the first appointment will allow dogs to be at their 'average' pain level, alternatively taking the mean of two or more baseline measurements before the intervention alleviates RTM effects (Brown et al., 2008; and Friedman et al., 2015). The study’s relatively small differences between treatment and control may be an artefact of the failure to follow scale guidelines or other test–retest reliability concerns. In addition, there is no discussion of language impacts on reliability and validity (Essner et al., 2020). The CBPI was scaled in English, but Corral et al. (2021) administered it to owners of various  nationalities. Furthermore, no information was provided on the veterinary clinical assessments (VCAs) carried out by different veterinarians at different sites and across different countries, raising additional questions about the reliability and validity of the outcome measures of the VCAs. A final issue is that numbers reported in charts and in-text descriptions occasionally did not match. Due to these concerns, confidence in the results is low.

Krautmann et al. (2021) investigated the safety of 7 monthly subcutaneous injections with bedinvetmab at different dose levels in healthy, mature laboratory dogs compared to a placebo. This study was randomised, controlled, and blinded. The authors do not describe randomised allocation, and the blinding of outcome assessors was incomplete. Sample sizes were small and were further reduced in each of the three studies in this preclinical trial, limiting statistical power and potentially impacting the detection of adverse health effects (AHEs). The sample population consisted of healthy, young dogs, limiting generalisability to sensitive subpopulations, such as older dogs with OA. However, this is the statutory safety protocol for licensing new drugs (EMEA VICH Topic GL43, 2008). Six randomised dogs were removed from the study subgroups during the acclimation period and replaced with dogs of the same sex. Four of those dogs were ‘deemed unsuitable’ with no further explanation for their removal or why replacement dogs were ‘deemed eligible’. It is difficult to determine what effect this replacement had on randomisation or magnitude, or direction of effects.

Krautmann et al. (2021) state that the 2 week trial of short-term co-administration of bedinvetmab and carprofen (NSAID) suggests that (a) a washout period between carprofen and bedinvetmab administration is not needed and that (b) intermittent short-term supplemental pain control via NSAIDs is possible. Study limitations suggest this statement should be viewed with caution.

Although rapidly progressive OA (RPOA) has not been reported in dogs, evidence from human clinical trials suggests a dose-dependent risk for developing RPOA with humanised anti-NGF mAb tanezumab both as monotherapy and when co-administered with NSAIDs (Schnitzer et al., 2015; and Hochberg et al., 2021).

Notably, the Food and Drug Administration (FDA) concluded that 2.5 mg tanezumab is not superior to oral prescription strength NSAIDs for alleviating pain and that adverse events such as accelerated joint destruction and abnormal peripheral sensation are more prevalent with tanezumab than NSAIDs (FDA, 2020; Hochberg et al., 2021; and Neogi et al., 2022). Due to these safety concerns, tanezumab has not been authorised (FDA, 2020; and EMA, 2021).

NSAIDs are a cornerstone for pain management of inflammatory conditions such as canine OA and demonstrate short-term efficacy (Hunt et al., 2015; and Hochberg et al., 2021). NSAID therapy can be associated with gastrointestinal, renal, and hepatic complications; their exact incidence rate is, however, unknown and, considering the frequent use of NSAIDs in clinical practice, likely low (Monteiro-Steagall et al., 2013; Hunt et al., 2015; and Gruen et al., 2022).

Studies by Lascelles et al. (2015) and Webster et al. (2014) have shown good pain relief with anti-NGF mAbs in dogs, and anecdotal evidence reveals a largely positive response of clients and veterinarians to the use of bedinvetmab for pain relief in clinical practice since its launch. This is promising and suggests that anti–NGF mAbs may be an additional choice for OA-related pain relief in dogs not tolerating NSAID treatment.

However, significant trial design limitations of the two reviewed studies in this Knowledge Summary mean that they provide only weak / inconclusive evidence for short-term safety and long-term efficacy of bedinvetmab. Additional well-designed studies examining the efficacy, effectiveness, and safety of short- and long-term treatment with bedinvetmab for dogs diagnosed with OA and chronic pain are needed.

## Methodology

**Search strategy:** 

Databases searched and covered	CAB Abstracts on OVID Platform (1973–2022 week 33) Web of Science Core Collection (1900–25/08/2022) PubMed accessed via NCBI platform (1910–25/08/2022)
Search strategy	CAB Abstracts: (dog or dogs or bitch* or canine).mp. (arthrit* or osteoarthrit* or osteo-arthrit* or OA or degenerative joint disease or DJD or joint disease).mp. (bedinvetmab or 'monoclonal antibody' or 'nerve growth factor' or NGF).mp. (safety or efficacy).mp. (pain or analgesia).mp. 1 AND 2 AND 3 AND 4 AND 5 Web of Science: (dog OR dogs OR bitch* OR canine) AND (arthrit* OR osteoarthrit* OR osteo–arthrit* OR OA OR ‘degenerative joint disease’ OR DJD OR ‘joint disease’) AND (bedinvetmab OR 'monoclonal antibody' OR 'nerve growth factor' OR NGF) AND (pain OR analgesia) PubMed: (dog OR dogs OR bitch* OR canine) AND (arthrit* OR osteoarthrit* OR osteo–arthrit* OR OA OR ‘degenerative joint disease’ OR DJD OR ‘joint disease’) AND (bedinvetmab OR 'monoclonal antibody' OR 'nerve growth factor' OR NGF) AND (pain OR analgesia)
Dates searches performed	25 Aug 2022

**Exclusion / Inclusion Criteria:** 

Exclusion:	Pre-defined exclusion criteria: conference articles, review articles, editorial materials, letters. Studies not focusing on the safety or efficacy of bedinvetmab for the treatment of canine osteoarthritis.
Inclusion:	Clinical trials in which the safety or efficacy of bedinvetmab for the treatment of canine osteoarthritis was studied.

**Search Outcome:** 

Database	Number of results	Excluded – Not answering PICO question	Excluded – Conference articles, review articles, editorial materials, letters	Total relevant papers
CAB Abstracts	7	4	2	1
Web of Science	30	22	6	2
PubMed	18	16	0	2
Total relevant papers when duplicates removed				2

## Acknowledgements

The author would like to extend thanks to Louise Buckley for the introduction to evidence-based veterinary medicine, and to Maureen O’Mara for her guidance.

## ORCID

Katrin Kronenberger: https://orcid.org/0000-0003-2717-6009

## Conflict of Interest

The author declares no conflicts of interest.

## References

[BIBR-1] Barnett A., Pols J., Dobson A. (2005). Regression to the mean: what it is and how to deal with it. International Journal of Epidemiology.

[BIBR-2] Brown D., Boston R., Coyne J., Farrar J. (2007). Development and psychometric testing of an instrument designed to measure chronic pain in dogs with osteoarthritis. American Journal of Veterinary Research.

[BIBR-3] D. Brown, R. Boston, J. Coyne, J Farrar (2008). Ability of the Canine Brief Pain Inventory to detect response to treatment in dogs with osteoarthritis. Journal of the American Veterinary Medical Association.

[BIBR-4] Brown D., Bell M., Rhodes L. (2013). Power of treatment success definitions when the Canine Brief Pain Inventory is used to evaluate carprofen treatment for the control of pain and inflammation in dogs with osteoarthritis. American Journal of Veterinary Research.

[BIBR-5] Corral M., Moyaert H., Fernandes T., Escalada M., Tena J., Walters R., Stegemann M. (2021). A prospective, randomized, blinded, placebo–controlled multi–site clinical study of bedinvetmab, a canine monoclonal antibody targeting nerve growth factor, in dogs with osteoarthritis. Veterinary Anaesthesia and Analgesia.

[BIBR-6] Donders A., Heijden G., Stijnen T., Moons K. (2006). Review: A gentle introduction to imputation of missing values. Journal of Clinical Epidemiology.

[BIBR-7] Agency European Medicines (2021). Raylumis. https://www.ema.europa.eu/en/medicines/human/EPAR/raylumis.

[BIBR-8] GL43 E.M.E.A.V.I.C.H.Topic (2008). Guidance for Industry. Target Animal Safety for Veterinary Pharmaceutical Products. The European Agency for the Evaluation of Medicinal Products. https://www.fda.gov/media/70438/download.

[BIBR-9] Enomoto M.Mantyth, P. Murrell, J. Innes, Lascelles B. (2019). Anti-nerve growth factor monoclonal antibodies for the control of pain in dogs and cats. Veterinary Record.

[BIBR-10] Essner A., Högberg H., Zetterberg L., Hellström K., Sjöström R., Gustås P. (2020). Investigating the Probability of Response Bias in Owner-Perceived Pain Assessment in Dogs with Osteoarthritis. Topics in Companion Animal Medicine.

[BIBR-11] Tanezumab F.D.A. (2020). FDA Efficacy Review. https://www.fda.gov/media/146924/download.

[BIBR-12] Friedman L., Furberg C., DeMets D., Reboussin D., Granger C., Friedman L., Furberg C., DeMets D., Reboussin D., Granger C. (2015). Baseline assessment. Fundamentals of Clinical Trials.

[BIBR-13] Gruen M., Lascelles B., Colleran E., Gottlieb A., Johnson J., Lotsikas P., Marcellin-Little D., Wright B. (2022). 2022 AAHA Pain Management Guidelines for Dogs and Cats. Journal of the American Animal Hospital Association.

[BIBR-14] Hochberg M., Carrino J., Schnitzer T., Guermazi A., Walsh D., White A., Nakajo S., Fountaine R., Hickman A., Pixton G., Viktrup L., Brown M., West C., Verburg K. (2021). Long-Term Safety and Efficacy of Subcutaneous Tanezumab Versus Nonsteroidal Antiinflammatory Drugs for Hip or Knee Osteoarthritis: A Randomized Trial. Arthritis & Rheumatology.

[BIBR-15] Hunt J., Dean R., Davis G., Murrell J. (2015). An analysis of the relative frequencies of reported adverse events associated with NSAID administration in dogs and cats in the United Kingdom. The Veterinary Journal.

[BIBR-16] Krautmann M., Walters R., Cole P., Tena J., Bergeron M., Messamore J., Mwangi D., Rai S., Dominowski P., Saad K., Zhu Y., Guillot M., Chouinard L. (2021). Laboratory safety evaluation of bedinvetmab, a canine anti-nerve growth factor monoclonal antibody, in dogs. The Veterinary Journal.

[BIBR-17] B. Lascelles, Knazovicky D., Case B., Freire M., Innes J., Drew A., Gearing D. (2015). A canine-specific anti-nerve growth factor antibody alleviates pain and improves mobility and function in dogs with degenerative joint disease-associated pain. BMC Veterinary Research.

[BIBR-18] Monteiro-Steagall B., Steagall P., Lascelles B. (2013). Systematic review of Nonsteroidal Anti-Inflammatory Drug-Induced Adverse Effects in Dogs. Journal of Veterinary Internal Medicine.

[BIBR-19] Neogi T., Hunter D., Churchill M., Shirinsky I., White A., Guermazi A., Omata M., Fountaine R., Pixton G., Viktrup L., Brown M., West C., Verburg K. (2022). Observed efficacy and clinically important improvements in participants with osteoarthritis treated with subcutaneous tanezumab: results from a 56-week randomized NSAID-controlled study. Arthritis Research & Therapy.

[BIBR-20] Schnitzer T., Ekman E., Spierings E., Greenberg H., Smith M., Brown M., West C., Verburg K. (2015). Efficacy and safety of tanezumab monotherapy or combined with non–steroidal anti–inflammatory drugs in the treatment of knee or hip osteoarthritis pain. Annals of the Rheumatic Diseases.

[BIBR-21] Webster R., G. Anderson, Gearing D. (2014). Canine Brief Pain Inventory scores for dogs with osteoarthritis before and after administration of a monoclonal antibody against nerve growth factor. American Journal of Veterinary Research.

