# Erratum to: In dogs diagnosed with osteoarthritis, how safe and effective is long-term treatment with bedinvetmab in providing analgesia?

**DOI:** 10.18849/ve.v8i3.668

**Published:** 2023-09-22

**Authors:** Katrin Kronenberger

**Keywords:** CANINE, OSTEOARTHRITIS, BEDINVETMAB, MONOCLONAL ANTIBODY, CANINE NERVE GROWTH FACTOR, ANALGESIA, CHRONIC OSTEOARTHRITIS ASSOCIATED PAIN, SAFETY; EFFECTIVENESS

## Abstract

The original article was published in Veterinary Evidence Vol 8, Issue 1 (2023): In dogs diagnosed with osteoarthritis, how safe and effective is long-term treatment with bedinvetmab in providing analgesia?

To aid interpretation, certain statements in the Knowledge Summary have been clarified. Refer to the full text and PDF for these amendments and corrections.

## Erratum

To aid interpretation, certain statements in the Knowledge Summary have been clarified. Refer below for these amendments and corrections.

**Clinical bottom line** Original: 'A single study suggests that treatment with bedinvetmab is effective. Two studies support the drug having few AHEs. Both studies have significant design limitations preventing the evaluation of bedinvetmab effectiveness. There is weak / inconclusive evidence for long-term efficacy and short-term safety of OA treatment with bedinvetmab. The decision to use bedinvetmab remains dependent on the judgement and experience of the clinician.'

Reason for change: The above conclusion in the published Knowledge Summary attributes the weak or inconclusive quality of evidence to a lack of support for the long-term effectiveness or safety of bedinvetmab. The level of evidence describes the confidence in the outcome not whether, in this case, the drug is efficacious or safe. For clarity, the Clinical bottom line has been amended.

Amended to: 'The quality of the published evidence available to answer the PICO 'In dogs diagnosed with osteoarthritis, how safe and effective is long-term treatment with bedinvetmab in providing analgesia' is weak due to design limitations of the two studies so far published. The decision to use bedinvetmab remains dependent on the judgement and experience of the clinician.'

**The evidence** Original: 'The authors neither describe the process of random assignment to the three study groups, nor further assignment to the smaller subgroups within these separate studies.'

Expanded to include: 'However, the authors make a general statement at the beginning of the methods section that good laboratory practice guidelines were followed.'

**The evidence** Original: 'The small cell sizes (n = 8) within each study condition raise concerns of overall potential sample bias or cell-specific sample bias.'

Expanded to include: 'Sample size of licensing studies is covered in internationally harmonised recommendations which try to balance risks of small sample size leading to potential bias with minimising the use of experimental animals (3Rs).'

**The evidence** Original: 'Generalising from mature laboratory beagles to an older target OA population may be a concern, although this is how most other analgesics are tested.'

Amended to: 'Whilst safety studies are designed as far as possible to be applicable across the whole population, the genetic diversity, intercurrent disease and other drugs patients may be receiving cannot be covered. For these reasons, field studies are conducted and ongoing adverse event reporting instituted to identify subpopulations that may be at risk.'

**The evidence** Original: 'Corral et al. (2021) did not follow this guidance to use the second pain score as baseline, which may have resulted in treatment and control groups being significantly different.'

Amended to: 'It is unclear whether Corral et al. (2021) followed the guidance to use the second pain score as baseline, which could result in treatment and control groups being significantly different.'

**The evidence** Original: 'Limitations of study design and execution of both studies suggest there currently is only weak evidence for long-term efficacy and short-term safety of bedinvetmab for the alleviation of OA-related pain in dogs.'

Amended to: 'The quality of the published evidence available to answer the PICO question is weak due to design limitations of the two studies so far published. Further studies are required to better understand long-term efficacy and short-term safety across the patient population.'

### Summary of the evidence Corral et al. (2021)

**Sample size** Original: 'Reported numbers of dogs in both phases of the study do not match reported number of dogs at start and after rescue removal.'

Amended to: 'Reported numbers of dogs in both phases of the study do not seem to match reported number of dogs at start and after rescue removal.'

**Intervention details** Original: '9% saline was administered SC at a dose volume equivalent to bedinvetmab administered monthly for 3 months.'

Reason for change: 9% is an error and should have been 0.9%. This error was introduced during the typesetting stage and is not an author error.

Corrected to: '0.9% saline was administered SC at a dose volume equivalent to bedinvetmab administered monthly for 3 months.'

**Main findings** Original: 'A significantly greater proportion of dogs in the bedinvetmab group 58/133* (43.5%) achieved CBPI-based treatment success versus placebo group 22/137* (16.9%) on day 28 (P = 0.0017). The difference between the groups is statistically significant (the null hypothesis that treatment with bedinvetmab is no different to treatment with placebo can be rejected), yet the mean difference in response rates between groups is small.'

Reason for change: Describing the bedinvetmab treatment effect as 'small' is a subjective interpretation.

Amended to: 'A significantly greater proportion of dogs in the bedinvetmab group 58/133* (43.5%) achieved CBPI-based treatment success versus placebo group 22/137* (16.9%) on day 28 (P = 0.0017). The difference between the groups is statistically significant (the null hypothesis that treatment with bedinvetmab is no different to treatment with placebo can be rejected). The clinical impact of the treatment success is difficult to assess in the absence of appropriate size estimates and confidence intervals. The difference on day 28 of the pain severity score (PSS) least squares mean is approximately 0.9 (on the 10 point scale) and of the pain interference score (PIS) approximately 1.2 (on the 10 point scale). Criteria for success for an individual compared to their baseline data was set at a reduction in PSS score of ≥ 1 and PIS of ≥ 2.'

**Limitations** Original: 'First test CBPI scores were used as baseline group comparison scores in violation of CBPI standard guidance on likely regression effects. Thus, subsequent repeated measures comparisons may be systematically distorted.'

To aid interpretation, the above statement has been clarified, and has now been amended to: 'It is unclear whether Corral et al. (2021) followed the CBPI guidance to use the second pain score as baseline. If the second score was not used, this could result in treatment and control groups being significantly different.'

**Limitations** Original: 'Removing randomised dogs' outcome data from the efficacy analysis may inflate the estimated treatment effect.'

Expanded to include: 'However, the potential effect of removing these data on the final analysis would not have changed the overall outcome.'

**Limitations** Original: 'Randomised dogs' outcome data were removed from the efficacy analysis but were included in safety analysis, which may lead to biased results of unknown direction. No explanation was provided for why this removal was only considered as treatment failure and not included in the efficacy analysis.'

Amended to: 'Randomised dogs' outcome data were removed from the efficacy analysis but were included in the safety analysis, which followed the study protocol but could lead to biased results of unknown direction.'

**Limitations** Original: 'No information was provided on how the dispenser's activity was isolated from veterinary staff or owners to ensure adequate blinding.'

Amended to: 'No information was provided on how the dispenser's activity was isolated from veterinary staff or owners, making it difficult to make an assessment of adequate blinding. However, the authors make a general statement in the methods section that good clinical practice guidelines were followed.'

**Limitations** Original: 'Different sections of the paper provide differing numbers of dogs for in comparison groups. It is difficult to determine which numbers are correct and whether p–values cited were based on correct or incorrect numbers of subjects.'

Amended to: 'Different sections of the paper provide differing numbers of dogs for in-comparison groups. This presents challenges in ascertaining the accuracy of whether cited p-values are derived from the correct number of subjects or if errors are present.'

### Summary of the evidence Krautmann et al. (2021)

**Intervention details** Original: '9% sterile saline solution for injection (Hospira, Inc.) administered SC at marked locations on the lateral neck and at volume equivalent to the 10 mg/kg dose volume.'

Reason for change: 9% is an error and should have been 0.9%. This error was introduced during the typesetting stage and is not an author error.

Corrected to: '0.9% sterile saline solution for injection (Hospira, Inc.) administered SC at marked locations on the lateral neck and at volume equivalent to the 10 mg/kg dose volume.'

**Outcome studied** Original: 'Study 1 Primary safety endpoint (objective): Pharmacokinetic profile of bedinvetmab: Mean bedinvetmab serum concentrations (μg/mL) after doses one and six, at three dose levels (1 mg/kg, 3 mg/kg, or 10 mg/kg SC); n = 8 per dose group).'

Reason for change: Describing pharmacokinetics as a primary safety endpoint is an error.

Corrected to: 'Study 1 Primary outcome (objective): Pharmacokinetic profile of bedinvetmab: Mean bedinvetmab serum concentrations (μg/mL) after doses one and six, at three dose levels (1 mg/kg, 3 mg/kg, or 10 mg/kg SC); n = 8 per dose group).'

**Limitations** Original: 'Enrolment of healthy, mature dogs at a single site: the sample population may not generalise to older dogs with OA.'

Amended to: 'Enrolment of healthy, mature dogs at a single site cannot evaluate potential risks across all subpopulations of patients that may receive bedinvetmab.'

**Limitations** Original: 'Allocation concealment is not described leaving the possibility of selection bias.'

Expanded to include: 'However, the authors make a general statement at the beginning of the methods section that good laboratory practice guidelines were followed.'

**Limitations** Original: 'Unclear result reporting.'

Amended to: 'Results reporting in some areas would benefit from clarification which would have aided assessment of the data.'

**Limitations** Original: 'Authors are all employees of the manufacturer of the drug, raising conflict of interest.'

Reason for change: The authors declared this conflict of interest. The statement has been amended and moved from the Limitations section to the Appraisal section.

Amended to: 'All authors in the Krautmann et al. (2021) study and the majority of authors in the Corral et al. (2021) study are employees of the manufacturer of the drug, which is acknowledged in the author list and declared in the conflict of interest statement.'

**Limitations** Original: '10x the recommended treatment dose is twice as much as recommended (EMEA VICH Topic GL43, 2008). '

This statement should have been in the Intervention details. In the Limitations section the statement has been amended to include: 'Although a 10x dose potentially increases subject risk, the lack of adverse events at this level of overdose supports the safety claims of the study.'

In the Intervention details section the statement has been amended to include: 'Bedinvetmab at 10mg/kg is 10x the recommended treatment dose which is twice as much as recommended (EMEA VICH Topic GL43, 2008).'

**Limitations** Original: 'Concurrent treatment increases difficulty identifying safety concerns related to intervention.'

Amended to: 'Whilst concurrent treatment increases difficulty identifying safety concerns related to the test drug i.e. did the test drug affect the side effect rate of the non-steroidal drug or vice versa; it was a stated aim of Study 3.'

**Limitations** Original: 'The significance level set at 10% together with small cell sizes increase the risk of incorrect rejection of the null hypothesis.'

Expanded to include: 'However, given the small cell size, this significance level is set to avoid missing a true treatment effect i.e. more sensitive but less specific to treatment effect.'

### Contribute to the Evidence

There are two main ways you can contribute to the evidence base while also enhancing your CPD:

- Tell us your information need
- Write a Knowledge Summary

Either way, you will be helping to add to the evidence base, and strengthen the decisions that veterinary professionals around the world make to give animals the best possible care. Learn more here: Author Hub

### Disclaimer

Knowledge Summaries are a peer-reviewed article type which aims to answer a clinical question based on the best available current evidence. It does not override the responsibility of the practitioner. Informed decisions should be made by considering such factors as individual clinical expertise and judgement along with patient's circumstances and owners' values. Knowledge Summaries are a resource to help inform and any opinions expressed within the Knowledge Summaries are the author's own and do not necessarily reflect the view of the RCVS Knowledge. Authors are responsible for the accuracy of the content. While the Editor and Publisher believe that all content herein are in accord with current recommendations and practice at the time of publication, they accept no legal responsibility for any errors or omissions, and make no warranty, express or implied, with respect to material contained within. For further information please refer to our Terms of Use .

